# Editorial on the Special Issue “Botulinum Toxin for the Treatment of Neurological Disorders: Where We Are and Where We Need to Go”

**DOI:** 10.3390/toxins14010041

**Published:** 2022-01-05

**Authors:** Mandar Jog, Alfonso Fasano

**Affiliations:** 1 Department of Clinical Neurological Sciences, London Health Sciences Centre—Lawson Health Research Institute, 339 Windermere Road, A10-026, London, ON N6A 5A5, Canada; 2 Schulich School of Medicine and Dentistry, University of Western, 1151 Richmond Street, London, ON N6A 3K7, Canada; 3 Movement Disorders Center, Toronto Western Hospital, University of Toronto, Toronto, ON M5T 2S8, Canada; alfonso.fasano@uhn.ca; 4 Krembil Brain Institute, Toronto, ON M5T 2S8, Canada

**Keywords:** botulinum toxins, movement disorders, unmet clinical needs, objective measurement, new indications

Over the past 30 years, botulinum toxin (BoNT) has seen an ever-expanding use in disorders afflicting the nervous system. Aside from the classical use of toxins for dystonia, its use for conditions such as spasticity and headache has become mainstream. In addition, technologies such as electromyography, ultrasound, and kinematics are reaching maturity for use in enhancing clinical assessment and injection. Finally, and equally importantly, newer toxins are potentially ready to reach the market over the next decade. Given these advances and this growth, we are pleased to introduce this Special Issue of *Toxins*, which includes 11 articles addressing the main theme of where we are (e.g., basic concepts, review articles of the current toxins, current indications) to where we are going (e.g., technologies, new indications, new research data, and new toxins).

Cervical dystonia is certainly one of the most established BoNT indications. The amount of evidence collected so far can now be condensed in meta-analysis and systematic reviews, such as the one published in this issue, which is specifically focused on abobotulinumtoxinA [1]. By gathering data on the clinical efficacy, patient-reported outcomes, safety data, and health economic outcomes from 6 placebo-controlled trials (8 articles), 6 active-controlled trials, and 16 observational studies (17 articles), this systematic review demonstrated that the routine use of abobotulinumtoxinA in cervical dystonia is well established, effective, and generally well tolerated, with a relatively low cost of treatment [1].

After decades of BoNT treatment, we are now adopting novel techniques for established indications. Fietzek and colleagues addressed the role of ultrasound for the personalized BoNT treatment of cervical dystonia, from basic technical considerations to muscle function and the anatomy of common cervical dystonic patterns. They also suggest a flow chart to achieve a personalized treatment of people with cervical dystonia [2]. Although usually safe and effective, BoNT is not always able to improve the functioning and quality of life in dystonia patients. We are also gaining a better understanding of the path of BoNT-resistant patients considering deep brain stimulation. Gorodetsky and colleagues found that of 67 patients referred for surgery, 49% eventually underwent it. Four (6%) patients were awaiting the procedure while the remaining 45% did not undergo deep brain stimulation for a variety of reasons, including patient refusal (53%), functional dystonia (20%), and—interestingly—the successful use of another BoNT (10%) in patients who had failed other types [3].

Spasticity is another established BoNT indication; our issue sets the stage with a review on muscle tone physiology and abnormalities [4] presenting data on the continuous increase in efficacy under repetitive injections of BoNT (five sessions every three months) in 24 adults with spastic foot drop followed over 390 days [5].

This issue also focused on recently approved BoNT indications, such as sialorrhea. Isaacson and colleagues reviewed the anatomy, function, and etiology of this disabling condition in Parkinson’s disease and provided newer evidence for the safety and efficacy of BoNT [6]. Headache is another approved and emerging BoNT treatment, as reviewed by Werner J. Becker [7]. Domínguez Vivero et al. took an additional step forward trying to understand what contributes to BoNT responsiveness in chronic migraine, finding that iron deposits in periaqueductal gray matter are associated with a poor response [8].

Finally, this issue directs its attention towards newer indications and BoNTs. The results of a randomized, double-blind, placebo-controlled study tested the hypothesis that customized IncobotulinumtoxinA injections for essential tremors are safe and effective were presented [9]. In this trial (NCT02207946), 30 patients were randomized and followed up to 24 weeks after treatment, overall finding that BoNT injections decreased tremor severity and improved hand motor function with a favorable tolerability profile [9]. This study also showed that tremor kinematic analytics technology could be successfully scaled for use in other clinical sites, a topic that the same group discussed in greater detail in another article of this issue [10].

Since its introduction as a treatment for strabismus, BoNT has had a phenomenal journey and is now recommended as first-line treatment for many indications and many others are currently being explored. This vitality is paralleled by an expansion of new BoNTs, as summarized by Choudhury and colleagues, who presented an overview of the toxo-pharmacology of conventional and novel BoNT preparations, including those awaiting imminent translation from the laboratory to the clinic [11].

In conclusion, this Special Issue of *Toxins* covers the past, present, and future of established and new BoNT formulations in approved and experimental indications, overall leaving us with the feeling that a significant amount of research has been carried out, and there will be abundant work in the near future aiming to improve the quality of life of our patients.
